# Serum Leptin Levels in Treatment-Naive Patients with Clinically Isolated Syndrome or Relapsing-Remitting Multiple Sclerosis

**DOI:** 10.1155/2014/486282

**Published:** 2014-11-19

**Authors:** Maria Eleftheria Evangelopoulos, Georgios Koutsis, Manolis Markianos

**Affiliations:** Department of Neurology, Eginition Hospital, Athens University Medical School, 74 Vassilisis Sophias Avenue, 11528 Athens, Greece

## Abstract

Several studies have investigated leptin levels in patients with multiple sclerosis (MS) with somewhat conflicting results. They have all focused on patients with established relapsing-remitting (RR) MS but have not specifically looked at patients with clinically isolated syndrome (CIS) suggestive of MS, in the early stages of disease. In this study, serum leptin levels were measured in 89 treatment-naïve patients with CIS (53 patients) or RRMS (36 patients) and 73 controls searching for differences between the groups and for associations with several disease parameters. The expected significant sexual dimorphism in leptin levels (higher levels in females) was observed in both MS patients and controls. Increased leptin levels were found in female patients with RRMS compared to female controls (*P* = .003) and female CIS patients (*P* = .001). Female CIS patients had comparable levels to controls. Leptin levels correlated positively to disease duration, but not to EDSS, in female patients with RRMS. The results of the present study do not indicate involvement of leptin in the early stages of MS. Normal leptin levels in patients with CIS suggest that leptin does not have a pathogenic role. The ratio leptin/BMI increases during disease course in female MS patients in a time-dependent and disability-independent manner.

## 1. Introduction

Leptin, a cytokine-like hormone released primarily from adipocytes, exhibits neuroendocrine properties influencing energy balance. Serum leptin levels regulate body weight by inhibiting food intake and stimulating energy expenditure and are higher in subjects with a high BMI and body fat [1, 2]. Additionally, leptin has immunomodulatory properties affecting neutrophil chemotaxis and macrophage proinflammatory cytokine expression. It also acts as a proinflammatory cytokine inducing a Th-1 immune response, thus playing a significant role in the pathogenesis of Th-1 mediated autoimmunity such as experimental autoimmune encephalomyelitis (EAE), an experimental model for multiple sclerosis (MS) [3]. In fact, leptin-deficient mice are resistant to the induction of EAE. On the other hand, activated T lymphocytes secrete leptin in active EAE brain lesions [4] indicating a role of leptin in the induction of EAE.

The role of leptin in MS pathogenesis has not yet been fully elucidated and reports have presented to some extent conflicting results. Early studies reported comparable serum leptin levels between relapsing-remitting (RR) MS patients and healthy subjects [5, 6]. Subsequently, Matarese et al. [7] found increased serum leptin in treatment-naïve RRMS patients. Higher serum leptin levels in treatment-naïve RRMS patients were also reported by Frisullo et al. [8]. Recently, two further studies have reported contradictory results. Emamgholipour et al. [9] found increased leptin levels in MS patients, whereas Rotondi et al. [10] found comparable levels with controls. Conflicting results between studies may be partly due to different sample sizes and different inclusion and exclusion criteria. Despite their differences, all studies to date have examined leptin levels in patients with established disease. These have been almost exclusively patients with clinically definite RRMS, with the exception of one study that also included patients with primary or secondary progressive MS [9]. None of these studies evaluated leptin levels at an early stage of the disease, in patients with clinically isolated syndrome (CIS) suggestive of MS, before the establishment of clinically definite MS.

The aim of the present study was to measure serum leptin levels in treatment-naive patients with CIS or RRMS and to evaluate their potential association with several disease parameters.

## 2. Subjects and Methods

Eighty-nine treatment-naïve patients (35 males, 54 females; age range from 20 to 45 years) recruited from year 2005 evenly throughout the years, as part of a larger study of CSF and serum neuroendocrine variables in MS and fulfilling the 2005 McDonald criteria for MS or possible MS were included in the study [11]. Fifty-three patients (21 males, 32 females) had CIS suggestive of MS while 36 (14 males, 22 females) had RRMS. Since there are no reports on serum leptin levels in early MS, patients at first episode (CIS) were separately evaluated in male and female subgroups. All patients had a good clinical status with low EDSS (range 0.0 to 4.0) and were drug-free at assessment, having never received any immunomodulatory therapy or corticosteroid treatment during previous relapse, as either the relapse was mild or underestimated by the patients. None of the patients had an endocrine or metabolic disease. At assessment, 54 patients (22 males) were in relapse [12] and 35 patients (13 males) in remission. Duration of illness ranged from 0.1 to 72 months for patients with CIS and from 2 to 192 months for RRMS patients. All patients were submitted to brain MRI and CSF analysis. Disability was assessed with the Expanded Disability Status Scale [13] (EDSS). Smoking history and numbers of cigarettes per day were registered.

A control group was built from 73 subjects in the same age range with patients, 30 males (age range 21 to 48 years) and 43 females (age range 18 to 48 years). Subjects, who formed part of the control group of a larger study of CSF and serum neuroendocrine variables in MS, were admitted to the hospital for diagnostic investigations and were found not to suffer from neurologic disease. More specifically, we included asymptomatic patients not only with positive serum treponemal tests, but also with negative CSF findings and a normal neurological and Mini Mental State Examination, as well as patients with a few nonspecific T2 lesions on brain MRI and a history atypical for MS, as well as normal neurological examination, cervical spine MRI, and CSF findings.

Written informed consent was obtained from all subjects and the study was approved by the Ethics Committee of Eginition Hospital, Athens University Medical School.

Body mass index (BMI, weight in kilograms divided by the square of height in meters) was calculated using self-reported height and weight. Blood samples were collected between 8.00 and 10.00 a.m. in all patients and controls, following an overnight fast. Serum was separated by centrifugation and kept at −30°C until estimation. Leptin is exceptionally stable in frozen serum samples, over two years at −20°C and over five freeze-thaw cycles [14]. Leptin was estimated in serum using the coated tube radioimmunoassay kit of DIAsource ImmunoAssays SA, Belgium. The manufacturer gives an analytical sensitivity of 0.1 ng/mL and intra- and interassay sensitivities close to 5%. We calculated an intra-assay coefficient of variation of 4.8 ± 3.8% (mean ± SD, range 0.06 to 11.5%) for 38 samples with leptin concentrations from 2.72 to 38.3 ng/mL. Both control and patient sera were included in every batch, to compensate for interassay variation.

Differences in leptin levels among groups were evaluated using analysis of variance (ANOVA) with covariates age and BMI. Differences in leptin levels normalized to BMI (leptin/BMI ratio) were also evaluated as previously suggested [7], using ANOVA with age as covariate. The leptin/BMI ratio can serve as a measure of adipocyte leptin secreting activity, since BMI correlates significantly with body fat [15]. Analyses were performed separately for males and females, since leptin levels are higher in females [1, 16]. Multiple regression analyses were performed with dependent variable serum leptin levels or leptin/BMI ratio and independent variables clinical and data. Linear regression was used in searching for correlations of leptin and leptin/BMI ratio to age, BMI, and duration of illness. For this analysis, data for duration of illness, expressed in months, were log-transformed to achieve a normal distribution (Shapiro-Wilk test for normality).

## 3. Results

Table 1 summarizes the clinical data for CIS and RRMS patients, both males and females.  Table 2 shows age, BMI, leptin levels, and leptin/BMI ratio in patients and controls. There were no significant differences in leptin or leptin/BMI ratio for the total group of patients (CIS + RRMS) when compared to controls, either for male or for female (Table 2). Significant differences were found when the three groups, controls, CIS, and RRMS, were compared for females. Patients with RRMS have higher values of both leptin and leptin/BMI ratio compared to controls and to female CIS. These differences were not significant for male patients.

Serum leptin levels and leptin/BMI ratios were higher in all female groups compared to all male groups. The known significant correlation between leptin and BMI was present in all groups, both male and female, with correlation coefficients ranging from 0.525 (CIS males) to 0.835 (RRMS females).

Age or smoking status had no effect on leptin or leptin/BMI ratio. No significant differences between groups were also found when patients were classified as active (patients who were in relapse or had GEL in MRI) and inactive (patients in remission without GEL in MRI). These variables were not introduced as independent variables in the following multiple regression analyses, in order to keep the number of independent variables low.

Multiple regression analyses were performed separately for male and female patients with dependent variable serum leptin levels or leptin/BMI ratio and independent variables duration of illness, relapse, oligoclonal bands in CSF, gadolinium enhancing lesions in MRI, and EDSS score. For male patients, no associations with any independent variable were found, either for leptin levels or leptin/BMI ratio (for leptin *R* = .2387, *F* = .35, and *P* = .88; for leptin/BMI ratio data are shown in Table 3). For females, no associations were found with relapse, presence of oligoclonal bands in CSF, gadolinium enhancing lesions in CSF, or EDSS score, but both leptin levels and leptin/BMI ratio were positively associated with the duration of illness (for leptin *R* = .4573, *F* = 2.54, *P* = .04, duration of illness beta = .395, and *P* = .006; for leptin/BMI ratio, see Table 3). This association though was present only for RR females, as revealed by performing multiple regressions separately for CIS and for RR patients (analytical data in Table 3).

**Figure 1:**
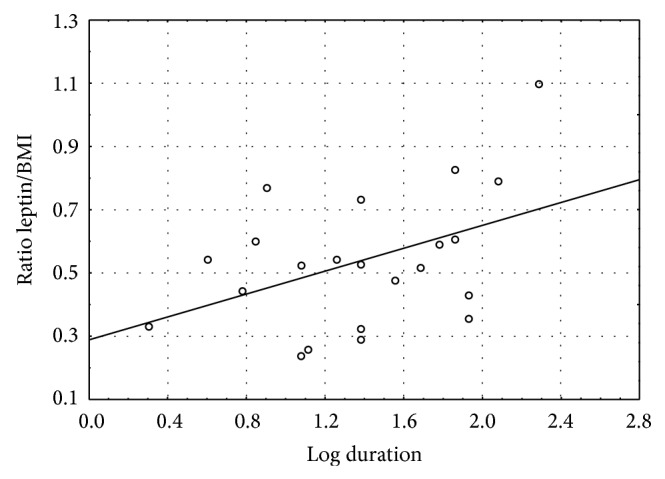
Correlation of the ratio leptin/BMI to duration of illness (in months, after logarithmic transformation) in female patients with relapsing-remitting MS (*n* = 22; *r* = .4368; *P* = .04).

The correlation between duration of illness and leptin/BMI ratio in female RR patients is depicted in Figure 1 (*n* = 22, *r* = .4368, and *P* = .041). The data for duration of illness were log-transformed to achieve a normal distribution. This correlation is not significant for male RR patients (*r* = −.0734, *P* = .80).

## 4. Discussion

The present study evaluated serum leptin levels not only in treatment-naïve patients with RRMS, but also in patients with CIS at the early stages of disease and examined potential associations with various disease parameters. Leptin levels were increased in female patients with RRMS and correlated positively to disease duration. On the other hand, female patients with CIS had normal leptin levels. No significant changes in leptin were found in male RRMS or CIS patients.

Leptin levels were strongly correlated to gender and BMI in both MS patients and controls. The sexual dimorphism in leptin levels (higher in females) is well established in normal subjects and has also been previously observed in MS patients [10, 16]. Regarding the correlation of leptin to BMI in MS patients specifically, previous reports have been somewhat conflicting. Matarese et al. [7] reported a loss of this correlation in MS patients with low disability in relapse. Rotondi et al. [10] reported that the correlation was maintained in MS patients with low disability in remission but lost in patients with high disability in remission. Our cohort included patients with low disability both in relapse and remission. Our findings are more in agreement with the second study, but this may be due to including our patients in remission.

Increased leptin levels in RRMS patients have been previously reported in several studies [7–9]. Gender specific analyses were reported in only one of these studies, which found increased levels in both female and male patients [8]. This is in contrast with our data that showed differences restricted to female patients. It is not easy to reconcile these conflicting findings, given that male sample size and percentage of males in relapse were very similar in both studies. A possible explanation for the discrepancy may lie in the fact that Frisullo et al. [8] included patients with previous corticosteroid treatment. Corticosteroid administration has been shown to increase serum leptin levels, albeit only transiently [10, 17].

Earlier studies that found no differences in leptin levels between MS patients and controls had lower patient numbers and one of them also included subjects on immunomodulatory therapy [5, 6]. The above may partly explain discrepancies with more recent reports, including the present findings. The only recent study that reported comparable leptin levels in patients and controls investigated exclusively patients in remission [10]. This may be offered as a possible explanation for the discrepancy, since the presence of relapse has been shown to significantly affect leptin levels in some studies. Batocchi et al. [5] found that leptin levels, measured before the first clinical relapse, during the follow-up period, were significantly higher compared to baseline values. Frisullo et al. [8] reported higher serum leptin levels in female RRMS patients in remission than in female RRMS patients in relapse and female controls. Nevertheless, we did not observe significant differences in leptin levels between patients in relapse and remission. However, we did observe a borderline significant negative correlation of leptin/BMI ratio in female CIS patients.

Several studies have shown that leptin leads to increased production of proinflammatory cytokines, upregulation of costimulatory molecules, increase in Th1 type of immune response, and suppression of Treg function [18]. Several lines of evidence also suggest that it has an important role in the induction and progression of EAE, the animal model of MS. Leptin-deficient mice are resistant to EAE induction and this can be reversed by the administration of leptin [19]. Accordingly, it has been suggested that the increase in leptin levels seen in patients with active RRMS might reflect a pathogenic role [20]. If such was the case, one would expect to see increased leptin levels at the early stages of MS, in patients with CIS. However, our study found no significant differences in leptin levels between patients with CIS and controls, suggesting that the hormone may not have a pathogenic role and that higher levels observed in RRMS patients may be a secondary phenomenon induced during the course of the disease. Beyond this, the lack of significant difference in leptin levels between male patients and controls also argues strongly against a pathogenic role.

In a recent study, by our group, female RRMS patients were found to have lower BMI compared to controls [21]. Since leptin levels are secreted in proportion to energy stored in body fat, acute BMI changes have been shown to affect circulating leptin concentrations [20]. One might expect, therefore, a reduction of leptin levels in female RRMS patients. Instead, we found disproportionately increased leptin levels, suggesting a possible dysregulation of the mechanisms regulating leptin secretion to body fat in MS patients.

Several studies investigated possible correlations between leptin levels and disease parameters, such as disease duration and EDSS, with negative results [5, 6, 10]. The present study, on the other hand, found a positive correlation between leptin levels and disease duration in female patients with RRMS. This correlation was also present for leptin/BMI ratio, further suggesting that adipocyte leptin secreting activity is increased in female patients as the disease progresses. However, the lack of association with EDSS indicates that changes in leptin secretion are not directly related to accumulating central nervous system damage. It is possible that the association of leptin with disease duration was not detected in previous studies because of smaller sample sizes [5] or less stringent exclusion criteria [6, 10]. More specifically, Chatzantoni et al. [6] also included patients on immunomodulatory treatment, whereas Rotondi et al. [10] also included patients that had received past corticosteroid treatment. We designed our study to include only treatment-naïve patients, both in terms of previous corticosteroid and immunomodulatory therapy. This greatly restricted the number of RRMS patients that could be included and that affected our sample size, but it may have allowed associations with surface that would otherwise be masked by confounding factors.

Several limitations that may influence the results of the present study, beyond the relatively restricted sample size, need to be mentioned. Firstly, the control group used was not healthy volunteers but subjects investigated as in-patients for possible neurological disease with negative findings, although, unlikely, this may have influenced serum leptin in controls. Secondly, the inclusion of only treatment-naïve RRMS patients means that the sample is not representative of the entire RRMS population. However, this avoids other potentially significant confounding factors. Finally, the use of self-reported BMI is a further limitation, although this applies to both patients and controls and taking also into account the high correlation between self-reported and measured values [22], it is not expected to significantly influence results.

## 5. Conclusion

The results of the present study on treatment-naïve patients with CIS and RRMS do not indicate the involvement of leptin in the early stages of the disease. The finding of increased leptin secretion only in female patients with RRMS, but not in patients at first demyelinating episode (CIS), suggests that leptin does not have a pathogenic role from the early stages of disease. The ratio leptin/BMI increases during disease course in female MS patients in a time-dependent and disability-independent manner.

## Figures and Tables

**Table 1:** Clinical data of male and female therapy-naïve patients with clinically isolated syndrome suggestive of MS (CIS) or with relapsing-remitting MS (RRMS).

	Males		Females	
	CIS	RRMS	CIS	RRMS
*N*	21	14	32	22
Duration, months	1.7 ± 2.6	56.8 ± 47.4	3.9 ± 13.1	45.1 ± 46.8
EDSS score	1.6 ± 1.2	2.3 ± 1.1	1.4 ± 1.1	1.7 ± 1.1
Smoking, no/yes	10/11	4/10	18/14	13/9
Relapse, no/yes	7/14	6/8	13/19	9/13
OB, no/yes	7/14	3/11	9/23	5/17
GEL, no/yes	11/10	7/7	16/16	5/17
Inactive/active	5/16	4/10	8/24	4/18

OB: oligoclonal bands in CSF; GEL: gadolinium enhanced lesions in MRI; inactive: patients in remission without GEL in MRI; active: patients in relapse or with GEL in MRI.

**Table 2:** Age, body mass index, serum leptin levels (ng/mL), and ratio leptin/BMI of controls and therapy-naïve patients with clinically isolated syndrome suggestive of MS (CIS) or relapsing-remitting MS (RRMS). Mean values ± SD. Evaluation by ANOVA with age and BMI as covariates for leptin and age as covariate for leptin/BMI ratio.

Group	*N*	Age	BMI	Leptin	Leptin/BMI
Males					
Controls	30	36.8 ± 8.1	25.7 ± 3.6	5.6 ± 3.5	0.21 ± 0.10
Patients, all	35	33.8 ± 6.3	25.4 ± 3.2	4.7 ± 1.6	0.18 ± 0.05
*F*/*P*				1.92/.17	1.64/.21
CIS	21	34.7 ± 6.2	26.1 ± 3.7	4.8 ± 1.8	0.18 ± 0.06
RRMS	14	32.6 ± 6.3	24.4 ± 1.9	4.5 ± 1.5	0.18 ± 0.05
*F*/*P*				1.26/.26	0.84/.44
Females					
Controls	43	33.6 ± 7.8	24.1 ± 3.8	10.9 ± 7.6	0.43 ± 0.25
Patients, all	54	32.9 ± 6.2	22.9 ± 4.3	10.6 ± 8.1	0.44 ± 0.25
*F*/*P*				1.29/.26	0.01/.92
CIS	32	33.3 ± 6.1	23.3 ± 4.4	9.2 ± 8.5	0.36 ± 0.26
RRMS	22	32.3 ± 6.5	22.3 ± 4.3	12.6 ± 7.2	0.54 ± 0.20
*F*/*P*				6.57/.002	3.54/.033
Females, planned comparisons					
Controls versus CIS, *F*/*P*				0.31/.58	1.48/.23
Controls versus RRMS, *F*/*P*				9.53/.003	2.98/.09
CIS versus RRMS, *F*/*P*				11.70/.001	7.07/.009

**Table 3:** Multiple regression analyses with dependent variable ratio leptin/BMI, an index of adipocyte leptin secreting activity in therapy-naïve male and female MS patients. CIS: clinically isolated syndrome; RR: relapsing-remitting MS; duration of illness in months; OB: oligoclonal bands in CSF; GEL: gadolinium enhanced lesions in MRI; EDSS: score in the expanded disability status scale.

	Males		Females, all		CIS-females		RR-females	
*R*	.2551		.4503		.3881		.6693	
*F*	0.40		2.44		0.92		2.60	
*P*	.84		.047		.748		.06	

Variable	Beta	*P*	Beta	*P*	Beta	*P*	Beta	*P*

Duration	−0.070	.71	0.394	**.007**	−0.230	.25	0.671	**.004**
Relapse	−0.1796	.36	−0.189	.29	−0.449	.09	0.159	.56
OB	−0.014	.94	0.024	.86	.108	.61	−0.054	.78
GEL	0.230	.23	−0.101	.49	−0.096	.65	−0.176	.42
EDSS	0.001	.99	0.148	.39	0.295	.22	−0.222	.41
